# Serum Concentrations of Endothelin-1 and Matrix Metalloproteinases-2, -9 in Pre-Hypertensive and Hypertensive Patients with Type 2 Diabetes

**DOI:** 10.3390/ijms17081182

**Published:** 2016-08-01

**Authors:** Krasimir Kostov, Alexander Blazhev, Milena Atanasova, Anelia Dimitrova

**Affiliations:** 1 Department of Physiology and Pathophysiology, Medical University-Pleven, 1 Kliment Ohridski Str., 5800 Pleven, Bulgaria; anelija.dimitrova@gmail.com; 2 Division of Biology, Medical University-Pleven, 1 Kliment Ohridski Str., 5800 Pleven, Bulgaria; yalishanda9@gmail.com (A.B.); milenaar2001@yahoo.com (M.A.)

**Keywords:** pre-hypertension, type 2 diabetes, endothelin-1, matrix metalloproteinases-2, matrix metalloproteinases-9, vascular remodeling

## Abstract

Endothelin-1 (ET-1) is one of the most potent vasoconstrictors known to date. While its plasma or serum concentrations are elevated in some forms of experimental and human hypertension, this is not a consistent finding in all forms of hypertension. Matrix metalloproteinases -2 and -9 (MMP-2 and MMP-9), which degrade collagen type IV of the vascular basement membrane, are responsible for vascular remodeling, inflammation, and atherosclerotic complications, including in type 2 diabetes (T2D). In our study, we compared concentrations of ET-1, MMP-2, and MMP-9 in pre-hypertensive (PHTN) and hypertensive (HTN) T2D patients with those of healthy normotensive controls (N). ET-1, MMP-2, and MMP-9 were measured by ELISA. Concentrations of ET-1 in PHTN and N were very similar, while those in HTN were significantly higher. Concentrations of MMP-2 and MMP-9 in PHTN and HTN were also significantly higher compared to N. An interesting result in our study is that concentrations of MMP-2 and MMP-9 in HTN were lower compared to PHTN. In conclusion, we showed that increased production of ET-1 in patients with T2D can lead to long-lasting increases in blood pressure (BP) and clinical manifestation of hypertension. We also demonstrated that increased levels of MMP-2 and MMP-9 in pre-hypertensive and hypertensive patients with T2D mainly reflect the early vascular changes in extracellular matrix (ECM) turnover.

## 1. Introduction

Endothelin-1 is one of the most potent vasoconstrictors known in humans to date [1]. Although, different types of cells, including cardiac myocytes, vascular smooth muscle cells (VSMCs), fibroblasts, or epithelial cells are able to synthesize and release ET-1, the most important biological source is the vascular endothelium [2]. ЕТ-1 is secreted primarily from the endothelial cells and influenced of the underlying VSMCs. Considering that approximately 80% of the total amount of ET-1 synthesized by endothelial cells is released toward the basolateral side of cells, tissue levels are higher than plasma levels. Thus, ET-1 acts primarily as a paracrine/autocrine peptide, and not as a circulating hormone [3]. Except through impact on vascular tone, ET-1 is involved in the complex regulation of BP through effects on renal hemodynamics and water-salt balance, influence on adrenal aldosterone, and catecholamine production, it also participates in the central and baroreceptor regulation and has positive inotropic effects on the heart [4]. In addition, ET-1 potentiates the action of other vasoconstrictors, such as angiotensin II (Ang II), phenylephrine, and serotonin [5].

The role of ET-1 and its receptors in the regulation of BP and in the pathogenesis of hypertension is not clearly established. For instance, while the plasma or vascular levels are elevated in some forms of experimental and human hypertension, this is not a consistent finding in all forms of hypertension [6,7]. Several reports suggested that patients with hypertension have elevated levels of ET-1. However, many other studies reported no difference of ET-1 levels between normotensive and hypertensive subjects [4]. An increased level of ET-1 has been demonstrated in some animal models of diabetes. Similarly, elevated levels of ET-1 have been reported in patients with diabetes, a finding not confirmed by all reports [8].

The role of MMPs in the process of vascular remodeling is extensively discussed [9,10,11,12]. Vascular remodeling is permanent process of structural changes in the vessel wall in response to hemodynamic stimulus [13]. In various forms of hypertension, including in human essential hypertension, resistance arteries undergoing inward remodeling, while larger vessels show hypertrophy [14,15,16]. MMP-2 and MMP-9, which degrade type IV collagen of the vascular basal membrane [17] are between most investigated MMPs and they play an essential role in the remodeling process [12,18,19]. MMP-2 and MMP-9 are secreted by a variety vascular and non-vascular cell types, such as endothelial cells, podocytes, fibroblasts, and myofibroblasts, macrophages formed by monocytes, as well as by resident tissue macrophages [20]. They are responsible for vascular remodeling, angiogenesis, inflammation, and atherosclerotic complications [21]. MMP-2 may participate in the pathogenesis of hypertension and through direct interaction with vasoactive peptides. For example, it could cleave big ET-1 to active ET-1, which have higher vasoconstrictor activity [22].

In patients with hypertension, it has been found that the plasma levels and activity of MMP-2 and MMP-9 can be increased [23,24,25], decreased [26,27,28], or unchanged [13,29,30]. The difficulty in finding precise correlations between activity/levels of ММРs in hypertension may depend largely on the impact of antihypertensive medications [31], as well on the clinical stage of patients who are assessed. Additionally, high blood glucose levels in diabetics induces disregulation of the MMPs/TIMPs system, which significantly upsets the balance between synthesis and degradation of vascular extracellular matrix (ECM) [32,33].

Based on the above, we tested serum concentrations of ET-1, MMP-2, and MMP-9 in pre-hypertensive and hypertensive patients with T2D, to clarify if there link between their levels and BP values.

## 2. Results

### 2.1. Serum Concentrations of Endothelin-1 (ET-1) in the Groups

**Figure 1:**
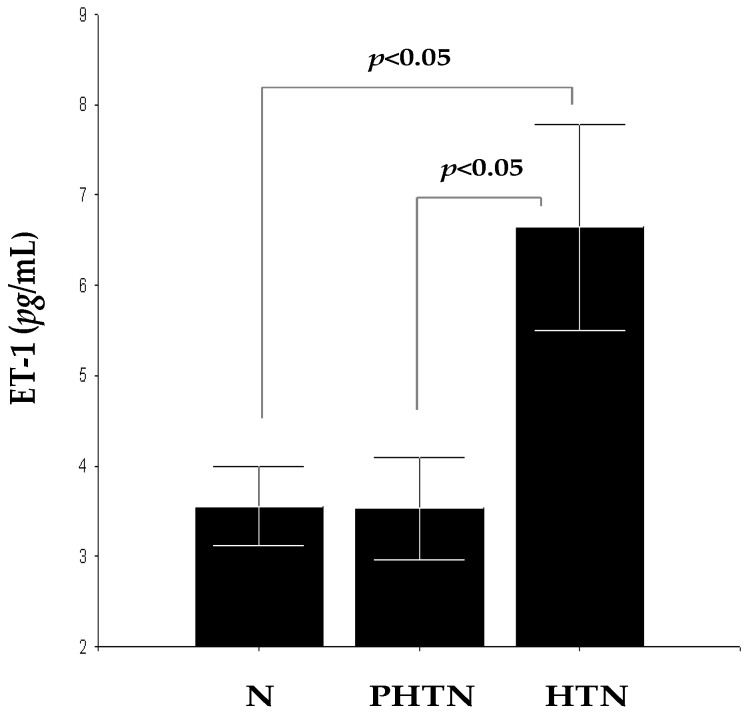
Serum concentrations of Endothelin-1 (ET-1) in pre-hypertensive (PHTN)/hypertensive (HTN) patients with T2D and healthy normotensive controls (N).

Concentrations of ET-1 were significantly higher in HTN 6.64 ± 5.36 pg/mL compared to PHTN 3.52 ± 2.29 pg/mL (*F* = 4.41, *p* < 0.05) and N 3.55 ± 1.78 pg/mL (*F* = 4.56, *p* < 0.05), but this difference was not observed between PHTN and N (*F* = 0.00, *p* > 0.05). Concentrations of ET-1 in PHTN and N are very similar, while those in HTN are significantly higher (Figure 1). These results show a possible connection between increased circulating ET-1 levels and clinical manifestation of arterial hypertension in patients with T2D. This is probably a consequence by dysmetabolic vascular changes leading to increased production of ET-1 and intensification of its pro-oxidant/pro-inflammatory effects and vasoconstrictor activity.

### 2.2. Serum Concentrations of Matrix Metalloproteinase-2 (MMP-2) in the Groups

**Figure 2:**
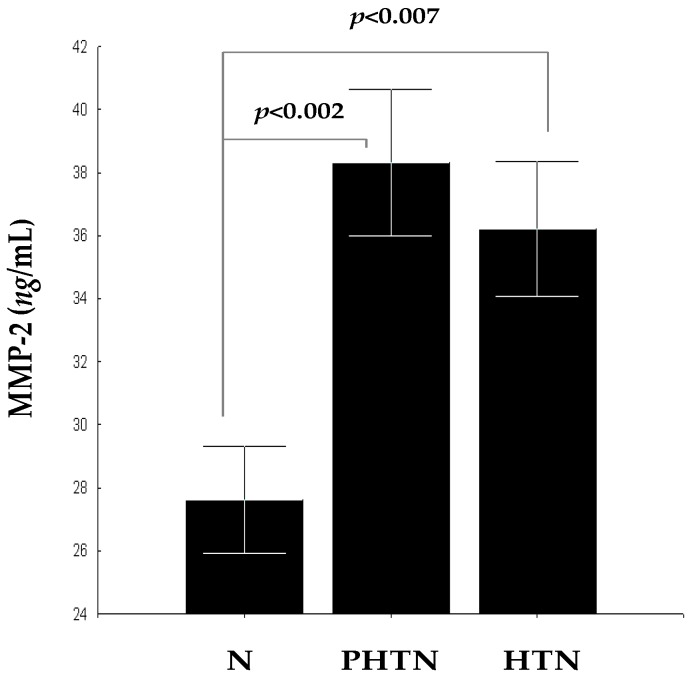
Serum concentrations of MMP-2 in PHTN, HTN, and N.

Concentrations of MMP-2 in PHTN 38.31 ± 9.12 ng/mL and HTN 36.22 ± 9.56 ng/mL were significantly higher compared to N 27.62 ± 6.94 ng/mL (*F* = 12.71, *p* < 0.002 and *F* = 8.41, *p* < 0.007) (Figure 2). Despite the fact that there were no statistical differences between PHTN and HTN (*F* = 0.39, *p* > 0.05), it is noteworthy that, in HTN, concentrations of MMP-2 are lower. This indicates that the balance between synthesis and degradation of ECM proteins in the vascular wall is developing dynamically over time. Probably, the expression of MMP-2 is induced at the beginning of the hypertensive process and its increased levels are mainly reflecting the early changes in ECM vascular turnover, provided that no significant vascular complications exist.

### 2.3. Serum Concentrations of MMP-9 in the Groups

**Figure 3:**
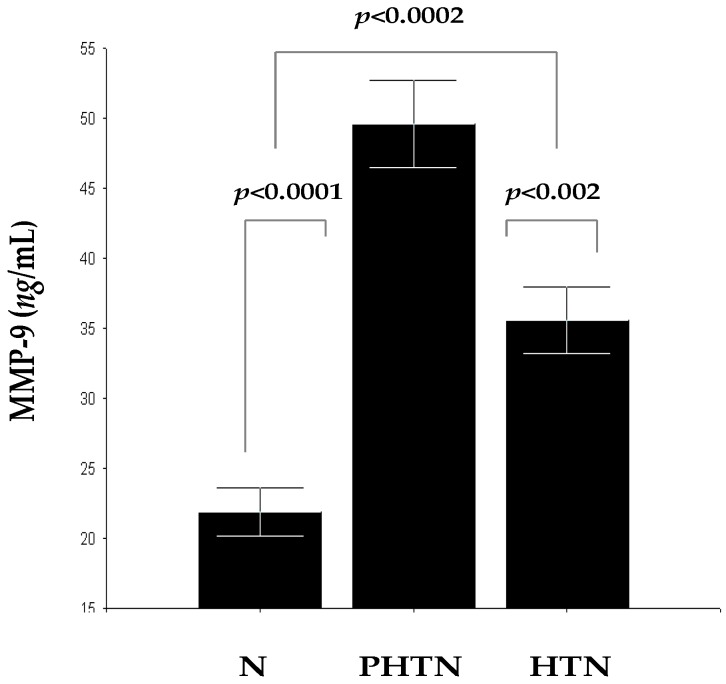
Serum concentrations of MMP-9 in PHTN, HTN, and N.

Concentrations of MMP-9 in PHTN 49.60 ± 12.37 ng/mL and HTN 35.55 ± 10.25 ng/mL were significantly higher compared to N 21.86 ± 7.47 ng/mL (*F* = 59.35, *p* < 0.0001 and *F* = 19.78, *p* < 0.0002). There were also statistical differences between PHTN and HTN, as it should be noted that, in HTN, concentrations of MMP-9 were significantly lower (*F* = 11.95, *p* < 0.002) (Figure 3). MMP-9, similar to MMP-2 is induced at the early stages of hypertension, and this is probably favorable to alleviate the initial vascular tensile stress. Later, the effects of MMP-2 and MMP-9 are counterbalanced by expression of tissue inhibitors of MMPs (TIMPs) and their concentrations began to decline.

## 3. Discussion

The results of our study demonstrate that concentrations of ET-1 in PHTN are very similar with those in N. This is not surprising, because elimination of ET-1 from the blood occurs rapidly. Additionally, the secretion of ET-1 from endothelial cells is polarized mainly toward the underlying VSMCs, which leads to a minimal increase of its circulating levels [6] in PHTN. On the contrary, it can be supposed that concentrations of ET-1 in HTN are significantly higher, which is supported by our experimental data. To exclude the influence of age and sex as factors in the analysis of the data is correct to clarify that concentrations of ET-1 do not show significant gender [34] and age differences. A number of studies of Donato et al., show that in healthy adults, plasma ET-1 concentrations either increase modestly or do not change with aging [35]. According to other authors, plasma ET-1 concentrations increase with age in some adults [36], as this process may be reversible after chronic exercise training [37]. Experimental data in rodent models do not show significant age-specific effects of ET-1 in relation to BP, because increased levels correlated with contractions in aortas from young rats, but not from old rats [38]. An age-associated increase in arterial pressure is a clinical hallmark of aging and results from joint effects of multiple factors, including, intimal-medial thickening, arterial pro-inflammatory responses, and vasoconstriction from Ang II and ET-1 effects [39]. Similar to our results, according to which plasma concentrations of ET-1 have been significantly higher in hypertensive patients with T1D and T2D compared to controls, are reported by Schneider et al. [40]. In keeping with this, it can be supposed that there is a possible connection between increased circulating levels of ET-1 and the development of hypertension in patients with T2D. This is probably the result of its enhanced vasoconstrictor, pro-oxidative, and pro-inflammatory action as a consequence of diabetes-related vascular changes. ET-1 is linked to the pathogenesis of hypertension by means of low-grade vascular inflammation [41,42] and oxidative stress at the vascular wall [43,44,45]. Low-grade inflammation localized in the vascular tissue is an important factor in the pathophysiology of hypertension [46]. ET-1 can activate the macrophages, which result in the release of pro-inflammatory and chemotactic mediators, such as TNF-α, IL-1, IL-6, and IL-8 [47,48,49]. Overexpression of ET-1 is associated with an inflammatory response, increased activation of NF-κB and the expression of several proinflammatory cytokines, such as TNF-α, IL-1, and IL-6 [50]. In turn, this transcription factor, and pro-inflammatory cytokines, can stimulate the production of ET-1 [51], and this could lead to increased BP [52,53,54]. The relationship between oxidative stress in the vessel wall and the development of hypertension is shown in many experimental models, including in human hypertension [55,56,57,58]. Various research supports the role of ET-1 in the formation of reactive oxygen species (ROS) and its relationship with oxidative stress and endothelial dysfunction in humans. ET-1 stimulates the production of ROS in human endothelial and vascular smooth muscle cell cultures [59,60], as well as in isolated vessels [61,62,63]. It is assumed that the main mechanism for the increased production of ROS in hypertension is increased expression of vascular NADPH oxidase [44,64,65,66]. Increased production of ROS in the vascular wall leads to activation of NF-κB. This, in turn, stimulates the synthesis of pro-inflammatory cytokines, chemokines, and adhesion molecules, which are associated with the development of vascular inflammatory response [67]. Thus, inflammation and oxidative stress form a vicious cycle in the development of endothelial dysfunction, which is implemented with active participation of ET-1. Elevated ET-1 levels may suppress NO synthesis in the endothelium [68]. One general observation, made in almost all studies, investigating endothelin receptor blockade and vascular function in animal models of hypertension, hypercholesterolemia, or atherosclerosis, is that long-term treatment with ETA receptor antagonists, improves endothelium-dependent NO-mediated vasodilation [69]. ET-1 causes insulin resistance and may participate in the pathogenesis of the metabolic syndrome [68]. Blockade of ET-1 signaling, improves vasodilation in diabetes and reduces insulin resistance [70]. Given the all vascular and extravascular effects of ET-1 taken together, we hypothesize that increased production of ET-1 in patients with T2D can lead to a long-lasting increase in blood pressure and clinical manifestation of hypertension.

In our study, we observed that, in PHTN, concentrations of MMP-2 and MMP-9 were significantly higher compared to N. Similar results are reported by Derosa et al., who found that the levels of MMP-2, MMP-9, and TIMP-1 are increased in patients with hypertension [23], as well as in patients with T2D [71]. Increased concentrations of MMP-9 in hypertensive patients with T2D have been reported earlier, also from other researchers [72,73,74]. An interesting result in our study is that concentrations of both metalloproteinases in HTN were also significantly higher compared to N, but lower compared to PHTN. In keeping with this, we hypothesize that MMP-2 and MMP-9 are induced at the early stages of the hypertension, and this is probably favorable for alleviation of the initial vascular tensile stress. Long-term effects of MMP-2 and MMP-9 in the vessel wall are counterbalanced by expression of TIMPs and their concentrations begin to decline [75,76,77], and may even be reduced in comparison to those of the control group. Reasons for this conclusion, given our data from a previous study which showed that the concentrations of MMP-9 in patients with mild, and especially with severe, hypertension are reduced significantly, compared to those of controls [78]. Similar results are reported by Zervoudaki et al., who observed a significant decrease of plasma levels of MMP-2 and MMP-9 in patients with essential hypertension in comparison with normotensive persons [28]. Li-Saw-Hee and coauthors also reported that the proteolytic activity of MMP-9 is suppressed in hypertensive patients [27]. The reduced concentration of MMP-9 in hypertension is associated with a decrease in the total activity of MMP-9, resulting in the accumulation of collagen in vascular wall of resistive arteries, reduction in their elasticity, and progression of hypertension [11]. Discrepancies between the data about MMP-2 and MMP-9 in different studies could be explained, considering that the balance between synthesis and degradation of ECM in hypertension is changing dynamically over time, and that production of ММРs are induced only for a certain period after the start of hypertension [76]. Elevated levels or activity of ММРs may indicate early changes in vascular ECM turnover, which later leads to the increase in arterial stiffness [58]. In the debut of hypertension, increased ММР expression is related to increasing arterial elasticity ex vivo [79] and in vivo [75]. Thus, the vascular wall normalizes the tensile stress and, in the short term, counteracts the increased BP. At this stage, MMPs are key players in ECM degradation. However, in the long-term, ECM proteins are synthesized again and they form new connections each other. This violates the beneficial effects of the initial remodeling process and ultimately leads to increased arterial stiffness [12]. It should be noted that, in comparison with the general population, atherosclerosis in patients with T2D is manifested earlier, and it may be more generalized and severe. Significantly higher serum concentrations of MMP-2 and MMP-9 are found in patients with T2D and atherosclerosis [74]. Overexpression of MMP-2 and MMP-9 in diabetic atherosclerotic plaques may increase their vulnerability which, in turn, increases the risk of ischemic cardiovascular events [80]. Furthermore, a chronic increase in MMP activation is central to age-associated arterial structural remodeling [81]. Throughout aging, the balance between MMPs and their inhibitors is changing [82] and MMP-2/-9 expression and activity are increased in vascular walls [83,84]. Typical vascular diseases such as hypertension and atherosclerosis also could be viewed as accelerated arterial aging and they are also linked to increased MMP activation [81]. Violation of the physiological balance between ММРs/TIMPs has been confirmed in early stages of diabetic retinopathy [73] and nephropathy [72,85]. Therefore, increased levels of MMP-2 and MMP-9 in pre-hypertensive and hypertensive patients with T2D, reflect mainly the early changes in ECM vascular turnover, provided that there are no significant vascular complications.

## 4. Materials and Methods

### 4.1. Study Population and Design

The study was approved by the University Ethics Committee and conducted in accordance with the Declaration of Helsinki. Written informed consent was obtained from all subjects. The study population was consisted of 60 persons: 40 prehypertensive and hypertensive T2D patients treated at the University Hospital Georgi Stranski, Pleven, and 20 healthy normotensive individuals. Three groups were formed: Group I (*n* = 20): normotensive controls (N); Group II (*n* = 20): pre-hypertension group (PHTN); and Group III (*n* = 20): hypertension group (HTN). Clinical characteristics of each group are shown in Table 1.

### 4.2. Immunological and Laboratory Testing

All laboratory determinations were obtained after a 12 h fast. To measure ET–1, MMP-2, MMP-9, and other laboratory parameters, blood was drawn into serum tubes. Serum was obtained after centrifugation at 1500 rpm for 15 min, and then stored at −80 °C until assayed.

#### 4.2.1. Immunological Testing

##### Indirect ELISA for Determination of ET-1

To measure ET-1, an ELISA kit from Biomedica Medizinprodukte GmbH and Co. KG, Divischgasse 4, A-1210 Wien, Austria (cat. No. BI-20052) was used. According to the manufacturer’s instructions, to each well-plate 50 µL tested sera or standard was added at various concentrations to construct a calibration curve. Then 200 µL of detection antibody was added to each well and incubated for 16–24 h at room temperature. After this period, plates were washed five times with 300 µL diluted wash buffer per well. After the last wash, 200 µL of conjugate was added to each well and incubated for 1 h at room temperature. The plate was washed again five times with 300 µL washing buffer, in each well 200 µL substrate was added and incubated for 30 min in the dark. The reaction was stopped with 50 µL of stop solution. The serum samples were assayed at 450 nm on an automatic micro-ELISA plate reader (Ceres UV 900 C, BioTek Instruments Inc., Winooski, VT, USA) at the Immunological Laboratory of Biology Department of Medical University, Pleven.

##### Indirect ELISA for Determination of MMP-2

To measure MMP-2, an ELISA kit from R and D Systems (cat. No. DMP2F0) (Minneapolis, MN, USA) was used. According to the manufacturer’s instructions, 100 μL of assay diluent RD1-74 was added to each well-plate , then 50 μL tested sera, diluted 1:10 with calibrator diluent RD5-32 (20 μL serum + 180 μL calibrator diluent) or standards was added at various concentrations to construct a calibration curve. After 2 h downtime at room temperature on a shaker, plates were washed three times with 400 μL wash buffer per well. After the last wash 200 μL of conjugate was added to each well and incubated for 2 h at room temperature on a shaker. The plate was washed again three times and in each well 200 μL substrate solution was added. This was incubated for 30 min at room temperature in the dark. The reaction was stopped with 50 μL of stop solution, and the color in the wells changed from blue to yellow. Within 30 min the serum samples were assayed at 450 nm on an automatic micro-ELISA plate reader (Ceres UV 900 C, BioTek Instruments Inc., Winooski, VT, USA) at the Immunological Laboratory of Biology Department of Medical University, Pleven.

##### Indirect ELISA for Determination of MMP-9

To measure MMP-9 an ELISA kit from R and D Systems (cat. No. DMP900) (Minneapolis, MN, USA) was used. According to the manufacturer’s instructions, to each well-plate 100 μL of assay diluent RD1-34 was added, then 100 μL tested sera, diluted 1:100 with calibrator diluent RD5-10 (10 μL serum + 990 μL calibrator diluent), or standards, was added at various concentrations to construct a calibration curve. After 2 h downtime at room temperature on a shaker, plates were washed three times with 400 μL wash buffer per well. After the last wash 200 μL anti-MMP-9 antibody conjugated with peroxidase and was incubated for 1 h at room temperature on a shaker. The plate was washed again three times was added and in each well 200 μL substrate solution was added. This was incubated for 30 min at room temperature in the dark. The reaction was stopped with 50 μL of stop solution, and the color in the wells changed from blue to yellow. Within 30 min the serum samples were assayed at 450 nm on an automatic micro-ELISA plate reader (Ceres UV 900 C, BioTek Instruments Inc., Winooski, VT, USA) at the Immunological Laboratory of Biology Department of Medical University, Pleven.

#### 4.2.2. Biochemical Assays

Enzymatic methods were used to measure of total cholesterol (TC), low-density lipoprotein cholesterol (LDL-C), high-density lipoprotein cholesterol (HDL-C), and triglyceride (TG). C-reactive protein (CRP) and glycated haemoglobin (HbA1c) were measured by a turbidimetric immunoassay.

### 4.3. Blood Pressure Classification and Measurements

#### 4.3.1. Blood Pressure Classification

The definitions of pre-hypertension (PHTN) and hypertension (HTN) were adopted according 2013 European Society of Cardiology (ESC)/ European Society of Hypertension (ESH) Hypertension Guidelines. PHTN, also known as high-normal BP was defined as systolic blood pressure (SBP) between 130 and 139 mmHg and/or diastolic blood pressure (DBP) between 85 and 89 mmHg. HTN was defined as SBP ≥ 140 mmHg and/or DBP ≥ 90 mmHg, or if the patients have been diagnosed or had taken antihypertensive drugs at any time during the preceding six months. Normal BP was defined as SBP between 120 and 129 mmHg and/or DBP between 80 and 84 mmHg.

#### 4.3.2. Blood Pressure Measurements

BP was measured using a standard cuff mercury sphygmomanometer on the left arm in a sitting position, after 5–10 min rest. All patients and control persons were subjected of three BP measurements. The average of the last two of three consecutive measurements was considered as the baseline BP.

### 4.4. Physical Measurements

Body mass index (BMI) was calculated, using the standard metric BMI formula (kg/m^2^). BMI between 18.5 and 24.9 was considered normal, 25 to 29.9 was considered overweight, and equal to or higher than 30 was considered obese.

### 4.5. Statistical Methods

Statgraphics Centurion XVI software (Statpoint Technologies, Inc., Warrenton, VA, USA) was used for statistical analyses. The significance of the differences between groups was assessed by Fisher’s *F*-test (ANOVA). The data are represented as means ± SD and *p* < 0.05 was considered statistically significant.

## 5. Conclusions

Hypertension is present in a high proportion of patients with T2D and enhances the risk of cardiovascular disease. Our results support a possible pathogenetic role of ET-1 in hypertension associated with T2D. We showed that increased serum concentrations of ET-1 in patients with T2D may assist clinical manifestation of hypertension. Endothelin receptor antagonists (ERAs) are a promising new and innovative drug class, which may have a particular role in the treatment of hypertension as part of the metabolic syndrome or T2D. We also demonstrated that increased concentrations of MMP-2 and MMP-9 in pre-hypertensive and hypertensive patients with T2D may indicate early changes in vascular ECM turnover which, over time, leads to the increase in arterial stiffness. Although, our findings should be confirmed in a larger prospective study, they have an important clinical implication, since it allows making an early assessment of patients with increased cardiovascular risk and allowing the earliest possible start of antihypertensive treatment, when the vascular remodeling process is still reversible. Further research is needed to investigate how glycemic control and antihypertensive drug therapy can affect concentrations of ET-1, MMP-2, MMP-9, and TIMPs, and what the relationship is of these molecules with the pathogenesis of hypertension in T2D.

## Figures and Tables

**Table 1:** Clinical characteristics of the groups in whole study population.

Variables All Groups (*n* = 60)	N	PHTN	HTN
	(*n* = 20)	(*n* = 20)	(*n* = 20)
Men, *n* (%)	9 (45.0)	7 (35.0)	8 (40.0)
Women, *n* (%)	11 (55.0)	13 (65.0)	12 (60.0)
Mean age, years ^1^	35.4 (19.0–56.0)	60.2 (46.0–79.0)	66.9 (45.0–89.0)
Duration of T2D ^1^	N/A ^2^	9.8 (1.0–34.0)	12.1 (2.0–22.0)
HbA1c (%) ^1^	N/A	7.0 (5.4–13.3)	8.0 (5.3–11.4)
BMI, kg/m^2^ ^1^	25.0 (22.0–28.0)	28.7 (24.0–35.0)	28.0 (24.0–34.0)
SBP, mmHg ^1^	119.0 (95.0–125.0)	136.6 (130.0–140.0)	156.7 (150.0–185.0)
DBP, mmHg ^1^	80.5 (70.0–85.0)	79.5 (70.0–90.0)	87.0 (75.0–100.0)
TC, mmol/L ^1^	3.9 (3.5–4.2)	5.3 (4.0–8.1)	5.2 (3.1–9.5)
LDL–C, mmol/L ^1^	2.5 (1.8–3.0)	3.3 (1.6–6.5)	3.0 (1.3–4.8)
HDL–C, mmol/L ^1^	1.2 (1.0–1.5)	0.9 (0.5–1.5)	0.9 (0.4–1.6)
TG, mmol/L ^1^	1.3 (1.2–1.5)	2.5 (1.3–4.8)	2.0 (1.0–4.0)
CRP, mg/L ^1^	1.1 (0.3–3.5)	8.0 (0.7–23.3)	8.3 (1.0–23.9)

^1^ Mean (range); ^2^ N/A, not available.

## Abbreviations

BP	Blood pressure
SBP	Systolic blood pressure
DBP	Diastolic blood pressure
T2D	Type 2 diabetes
ET-1	Endothelin-1
Ang II	Angiotensin II
MMP-2	Matrix metalloproteinase-2
MMP-9	Matrix metalloproteinase-9
MMPs	Matrix metalloproteinases
TIMPs	Tissue inhibitors of MMPs
VSMCs	Vascular smooth muscle cells
ECM	Extracellular matrix
PHTN	Pre-hypertension group
HTN	Hypertension group
N	Normotensive controls
ROS	Reactive oxygen species
NF-kB	Nuclear factor-kappa B
TNF-α	Tumor necrosis factor-alpha
IL-1	Interleukin-1
IL-6	Interleukin-6
IL-8	Interleukin-8

## References

[1] Meyers K.E., Sethna C. (2013). Endothelin antagonists in hypertension and kidney disease. Pediat. Nephrol..

[2] Rodríguez-Pascual F., Busnadiego O., Lagares D., Lamas S. (2011). Role of endothelin in the cardiovascular system. Pharmacol. Res..

[3] Del Villar C.P., Alonso C.J.G., Feldstein C.A., Juncos L.A., Romero J.C. (2005). Role of endothelin in the pathogenesis of hypertension. Mayo Clin. Proc..

[4] Kohan D.E., Rossi N.F., Inscho E.W., Pollock D.M. (2011). Regulation of blood pressure and salt homeostasis by endothelin. Physiol. Rev..

[5] Tostes R.C., Fortes Z.B., Callera G.E., Montezano A.C., Touyz R.M., Webb R.C., Carvalho M.H.C. (2008). Endothelin, sex and hypertension. Clin. Sci..

[6] Hynynen M.M., Khalil R.A. (2006). The vascular endothelin system in hypertension–recent patents and discoveries. Recent Pat. Cardiovasc. Drug Discov..

[7] Vorobiof G., Blaxall B.C., Bisognano J.D. (2006). The future of endothelin–receptor antagonism as treatment for systemic hypertension. Curr. Hypertens. Rep..

[8] Cardillo C., Campia U., Bryant M.B., Panza J.A. (2002). Increased activity of endogenous endothelin in patients with type II diabetes mellitus. Circulation.

[9] Dollery C.M., McEwan J.R., Henney A.M. (1995). Matrix metalloproteinases and cardiovascular disease. Circ. Res..

[10] Galis Z.S., Khatri J.J. (2002). Matrix metalloproteinases in vascular remodeling and atherogenesis the good, the bad, and the ugly. Circ. Res..

[11] Dhingra R., Pencina M.J., Schrader P., Wang T.J., Levy D., Pencina K., Vasan R.S. (2009). Relations of matrix remodeling biomarkers to blood pressure progression and incidence of hypertension in the community. Circulation.

[12] Lemarié C.A., Tharaux P.L., Lehoux S. (2010). Extracellular matrix alterations in hypertensive vascular remodeling. J. Mol. Cell. Cardiol..

[13] Friese R.S., Rao F., Khandrika S., Thomas B., Ziegler M.G., Schmid-Schönbein G.W., O’Connor D.T. (2009). Matrix metalloproteinases: Discrete elevations in essential hypertension and hypertensive end–stage renal disease. Clin. Exp. Hypertens..

[14] Heagerty A.M., Aalkjaer C., Bund S.J., Korsgaard N., Mulvany M.J. (1993). Small artery structure in hypertension. Dual processes of remodeling and growth. Hypertension.

[15] Mulvany M.J., Baumbach G.L., Aalkjaer C., Heagerty A.M., Korsgaard N., Schiffrin E.L., Heistad D.D. (1996). Vascular remodeling. Hypertension.

[16] Mulvany M.J. (2002). Small artery remodeling and significance in the development of hypertension. Physiology.

[17] Intengan H.D., Schiffrin E.L. (2001). Vascular remodeling in hypertension roles of apoptosis, inflammation, and fibrosis. Hypertension.

[18] Raffetto J.D., Khalil R.A. (2008). Matrix metalloproteinases and their inhibitors in vascular remodeling and vascular disease. Biochem. Pharmacol..

[19] Humphrey J.D. (2008). Mechanisms of arterial remodeling in hypertension coupled roles of wall shear and intramural stress. Hypertension.

[20] Bourboulia D., Stetler-Stevenson W.G. (2010). Matrix metalloproteinases (MMPs) and tissue inhibitors of metalloproteinases (TIMPs): Positive and negative regulators in tumor cell adhesion. Semin. Cancer Biol..

[21] Jaiswal A., Chhabra A., Malhotra U., Kohli S., Rani V. (2011). Comparative analysis of human matrix metalloproteinases: Emerging therapeutic targets in diseases. Bioinformation.

[22] Fernandez-Patron C., Radomski M.W., Davidge S.T. (1999). Vascular matrix metalloproteinase-2 cleaves big endothelin-1 yielding a novel vasoconstrictor. Circ. Res..

[23] Derosa G., D’Angelo A., Ciccarelli L., Piccinni M.N., Pricolo F., Salvadeo S., Cicero A.F. (2006). Matrix metalloproteinase-2, -9, and tissue inhibitor of metalloproteinase-1 in patients with hypertension. Endothelium.

[24] Tayebjee M.H., Nadar S.K., MacFadyen R.J., Lip G.Y. (2004). Tissue inhibitor of metalloproteinase-1 and matrix metalloproteinase-9 levels in patients with hypertension: Relationship to tissue doppler indices of diastolic relaxation. Am. J. Hypertens..

[25] Wallace S., McEniery C.M., Dakham Z., Pusalkar P., Maki-Petaja K., Ashby M.J., Wilkinson I.B. (2005). Matrix metalloproteinase-9 (MMP-9), MMP-2, and serum elastase activity are associated with systolic hypertension and arterial stiffness. Arterioscler. Thromb. Vasc. Biol..

[26] Intengan H.D., Schiffrin E.L. (1999). Collagen degradation is diminished in mesenteric arteries of spontaneously hypertensive rats after hypertension is established. Hypertension.

[27] Li–Saw–Hee F.L., Edmunds E., Blann A.D., Beevers D.G., Lip G.Y. (2000). Matrix metalloproteinase-9 and tissue inhibitor metalloproteinase-1 levels in essential hypertension: Relationship to left ventricular mass and anti-hypertensive therapy. Int. J. Cardiol..

[28] Zervoudaki A., Economou E., Stefanadis C., Pitsavos C., Tsioufis K., Aggeli C., Toutouzas P. (2003). Plasma levels of active extracellular matrix metalloproteinases 2 and 9 in patients with essential hypertension before and after antihypertensive treatment. J. Hum. Hypertens..

[29] Ahmed S.H., Clark L.L., Pennington W.R., Webb C.S., Bonnema D.D., Leonardi A.H., Zile M.R. (2006). Matrix metalloproteinases/tissue inhibitors of metalloproteinases: Relationship between changes in proteolytic determinants of matrix composition and structural, functional, and clinical manifestations of hypertensive heart disease. Circulation.

[30] Martinez M.L., Lopes L.F., Coelho E.B., Nobre F., Rocha J.B., Gerlach R.F., Tanus-Santos J.E. (2006). Lercanidipine reduces matrix metalloproteinase-9 activity in patients with hypertension. J. Cardiovasc. Pharmacol..

[31] Schieffer B., Bünte C., Witte J., Hoeper K., Böger R.H., Schwedhelm E., Drexler H. (2004). Comparative effects of AT1-antagonism and angiotensin-converting enzyme inhibition on markers of inflammation and platelet aggregation in patients with coronary artery disease. J. Am. Coll. Cardiol..

[32] Pugliese G., Pricci F., Pugliese F., Mene P., Lenti L., Andreani D., Di U.M. (1994). Mechanisms of glucose-enhanced extracellular matrix accumulation in rat glomerular mesangial cells. Diabetes.

[33] Death A.K., Fisher E.J., McGrath K.C., Yue D.K. (2003). High glucose alters matrix metalloproteinase expression in two key vascular cells: Potential impact on atherosclerosis in diabetes. Atherosclerosis.

[34] Suzuki N., Matsumoto H., Kitada C., Yanagisawa M., Miyauchi T., Masaki T., Fujino M. (1989). Immunoreactive endothelin-1 in plasma detected by a sandwich-type enzyme immunoassay. J. Cardiovasc. Pharmacol..

[35] Donato A.J., Gano L.B., Eskurza I., Silver A.E., Gates P.E., Jablonski K., Seals D.R. (2009). Vascular endothelial dysfunction with aging: Endothelin-1 and endothelial nitric oxide synthase. Am. J. Physiol. Heart Circ. Physiol..

[36] Seals D.R., Jablonski K.L., Donato A.J. (2011). Aging and vascular endothelial function in humans. Clin. Sci..

[37] Maeda S., Tanabe T., Miyauchi T., Otsuki T., Sugawara J., Iemitsu M., Matsuda M. (2003). Aerobic exercise training reduces plasma endothelin-1 concentration in older women. J. Appl. Physiol..

[38] Barton M., Cosentino F., Brandes R.P., Moreau P., Shaw S., Lüscher T.F. (1997). Anatomic heterogeneity of vascular aging role of nitric oxide and endothelin. Hypertension.

[39] Wang M., Zhang J., Telljohann R., Jiang L., Wu J., Monticone R.E., Lakatta E.G. (2012). Chronic matrix metalloproteinase inhibition retards age-associated arterial proinflammation and increase in blood pressure. Hypertension.

[40] Schneider J.G., Tilly N., Hierl T., Sommer U., Hamann A., Dugi K., Kasperk C. (2002). Elevated plasma endothelin-1 levels in diabetes mellitus. Am. J. Hypertens..

[41] Böhm F., Pernow J. (2007). The importance of endothelin-1 for vascular dysfunction in cardiovascular disease. Cardiovasc. Res..

[42] Pernow J., Shemyakin A., Böhm F. (2012). New perspectives on endothelin-1 in atherosclerosis and diabetes mellitus. Life Sci..

[43] Skalska A.B., Pietrzycka A., Stępniewski M. (2009). Correlation of endothelin-1 plasma levels with plasma antioxidant capacity in elderly patients treated for hypertension. Clin. Biochem..

[44] Romero M., Jiménez R., Sánchez M., López–Sepúlveda R., Zarzuelo A., Tamargo J., Duarte J. (2010). Vascular superoxide production by endothelin-1 requires Src non-receptor protein tyrosine kinase and MAPK activation. Atherosclerosis.

[45] Piechota A., Polańczyk A., Gorąca A. (2010). Role of endothelin-1 receptor blockers on hemodynamic parameters and oxidative stress. Pharmacol. Rep..

[46] Savoia C., Sada L., Zezza L., Pucci L., Lauri F.M., Befani A., Volpe M. (2011). Vascular inflammation and endothelial dysfunction in experimental hypertension. Int. J. Hypertens..

[47] Ruetten H., Thiemermann C. (1997). Endothelin-1 stimulates the biosynthesis of tumour necrosis factor in macrophages: ET-receptors, signal transduction and inhibition by dexamethasone. J. Physiol. Pharmacol..

[48] Hofman F.M., Chen P., Jeyaseelan R., Incardona F., Fisher M., Zidovetzki R. (1998). Endothelin-1 induces production of the neutrophil chemotactic factor interleukin-8 by human brain-derived endothelial cells. Blood.

[49] Browatzki M., Schmidt J., Kübler W., Kranzhöfer R. (2000). Endothelin–1 induces interleukin-6 release via acctivation of the transcription factor NF-κB in human vascular smooth muscle cells. Basic Res. Cardiol..

[50] Yang L.L., Gros R., Kabir M.G., Sadi A., Gotlieb A.I., Husain M., Stewart D.J. (2004). Conditional cardiac overexpression of endothelin-1 induces inflammation and dilated cardiomyopathy in mice. Circulation.

[51] Virdis A., Schiffrin E.L. (2003). Vascular inflammation: A role in vascular disease in hypertension. Curr. Opin. Nephrol. Hypertens..

[52] Vierhapper H., Wagner O., Nowotny P., Waldhäusl W. (1990). Effect of endothelin-1 in man. Circulation.

[53] Schneider M.P., Hilgers K.F., Klingbeil A.U., John S., Veelken R., Schmieder R.E. (2000). Plasma endothelin is increased in early essential hypertension. Am. J. Hypertens..

[54] Letizia C., Celi M., Cerci S., Scuro L., Delfini E., Subioli S., D’Erasmo E. (2001). High circulating levels of adrenomedullin and endothelin-1 in obesity associated with arterial hypertension. Ital. Heart J. Suppl..

[55] Vaziri N.D. (2004). Roles of oxidative stress and antioxidant therapy in chronic kidney disease and hypertension. Curr. Opin. Nephrol. Hypertens..

[56] Peterson J.R., Sharma R.V., Davisson R.L. (2006). Reactive oxygen species in the neuropathogenesis of hypertension. Curr. Hypertens. Rep..

[57] Harrison D.G., Gongora M.C. (2009). Oxidative stress and hypertension. Med. Clin. N. Am..

[58] Briones A.M., Touyz R.M. (2010). Oxidative stress and hypertension: Current concepts. Curr. Hypertens. Rep..

[59] Dong F., Zhang X., Wold L.E., Ren Q., Zhang Z., Ren J. (2005). Endothelin-1 enhances oxidative stress, cell proliferation and reduces apoptosis in human umbilical vein endothelial cells: Role of ETB receptor, NADPH oxidase and caveolin-1. Br. J. Pharmacol..

[60] Duerrschmidt N., Wippich N., Goettsch W., Broemme H.J., Morawietz H. (2000). Endothelin-1 induces NAD (P) H oxidase in human endothelial cells. Biochem. Biophys. Res. Commun..

[61] Galle J., Lehmann-Bodem C., Hübner U., Heinloth A., Wanner C. (2000). CyA and OxLDL cause endothelial dysfunction in isolated arteries through endothelin-mediated stimulation of O^2−^ formation. Nephrol. Dial. Transpl..

[62] Loomis E.D., Sullivan J.C., Osmond D.A., Pollock D.M., Pollock J.S. (2005). Endothelin mediates superoxide production and vasoconstriction through activation of NADPH oxidase and uncoupled nitric-oxide synthase in the rat aorta. J. Pharmacol. Exp. Ther..

[63] López–Sepúlveda R., Gómez-Guzmán M., Zarzuelo M.J., Romero M., Sánchez M., Quintela A.M., Duarte J. (2011). Red wine polyphenols prevent endothelial dysfunction induced by endothelin-1 in rat aorta: Role of NADPH oxidase. Clin. Sci..

[64] Mohazzab K.M., Kaminski P.M., Wolin M.S. (1994). NADH oxidoreductase is a major source of superoxide anion in bovine coronary artery endothelium. Am. J. Physiol. Heart Circ. Physiol..

[65] Kamata K., Kanie N., Matsumoto T., Kobayashi T. (2004). Endothelin-1-induced impairment of endothelium-dependent relaxation in aortas isolated from controls and diabetic rats. J. Cardiovasc. Pharmacol..

[66] Kanie N., Kamata K. (2002). Effects of chronic administration of the novel endothelin antagonist J-104132 on endothelial dysfunction in streptozotocin-induced diabetic rat. Br. J. Pharmacol..

[67] Camici G.G., Sudano I., Noll G., Tanner F.C., Lüscher T.F. (2009). Molecular pathways of aging and hypertension. Curr. Opin. Nephrol. Hypertens..

[68] Ergul A. (2011). Endothelin–1 and diabetic complications: Focus on the vasculature. Pharmacol. Res..

[69] Barton M. (2000). Endothelial dysfunction and atherosclerosis: Endothelin receptor antagonists as novel therapeutics. Curr. Hypertens. Rep..

[70] Matsumoto T., Lopes R.A., Taguchi K., Kobayashi T., Tostes R.C. (2014). Linking the beneficial effects of current therapeutic approaches in diabetes to the vascular endothelin system. Life Sci..

[71] Derosa G., D’angelo A., Tinelli C., Devangelio E., Consoli A., Miccoli R., Cicero A.F.G. (2007). Evaluation of metalloproteinase 2 and 9 levels and their inhibitors in diabetic and healthy subjects. Diabetes Metab..

[72] Ebihara I., Nakamura T., Shimada N., Koide H. (1998). Increased plasma metalloproteinase-9 concentrations precede development of microalbuminuria in non-insulin-dependent diabetes mellitus. Am. J. Kidney Dis..

[73] Giebel S.J., Menicucci G., McGuire P.G., Das A. (2005). Matrix metalloproteinases in early diabetic retinopathy and their role in alteration of the blood-retinal barrier. Lab. Investig..

[74] Santo Signorelli S., Malaponte G., Libra M., Di Pino L., Celotta G., Bevelacqua V., Pennisi G. (2005). Plasma levels and zymographic activities of matrix metalloproteinases 2 and 9 in type II diabetics with peripheral arterial disease. Vasc. Med..

[75] Jackson Z.S., Gotlieb A.I., Langille B.L. (2002). Wall tissue remodeling regulates longitudinal tension in arteries. Circ. Res..

[76] Flamant M., Placier S., Dubroca C., Esposito B., Lopes I., Chatziantoniou C., Lehoux S. (2007). Role of matrix metalloproteinases in early hypertensive vascular remodeling. Hypertension.

[77] Watts S.W., Rondelli C., Thakali K., Li X., Uhal B., Pervaiz M.H., Fink G.D. (2007). Morphological and biochemical characterization of remodeling in aorta and vena cava of DOCA-salt hypertensive rats. Am. J. Physiol Heart Circ. Physiol..

[78] Kostov K., Dimitrova A., Grigoryan A., Tisheva S., Ruseva A., Atanasova M., Gospodinov K., Blazhev A. (2014). Changes in the serum levels of endothelin-1, matrix metalloproteinases-2,-9 and c-reactive protein in patients with mild and severe degree of arterial hypertension. Cardiovasc. Res..

[79] Lehoux S., Lemarié C.A., Esposito B., Lijnen H.R., Tedgui A. (2004). Pressure-induced matrix metalloproteinase-9 contributes to early hypertensive remodeling. Circulation.

[80] Cipollone F., Iezzi A., Fazia M., Zucchelli M., Pini B., Cuccurullo C., Chiarelli F. (2003). The receptor RAGE as a progression factor amplifying arachidonate-dependent inflammatory and proteolytic response in human atherosclerotic plaques: Role of glycemic control. Circulation.

[81] Wang M., Kim S.H., Monticone R.E., Lakatta E.G. (2015). Matrix metalloproteinases promote arterial remodeling in aging, hypertension, and atherosclerosis. Hypertension.

[82] Harvey A., Montezano A.C., Touyz R.M. (2015). Vascular biology of ageing-Implications in hypertension. J. Mol. Cell. Cardiol..

[83] Monk B.A., George S.J. (2014). The effect of ageing on vascular smooth muscle cell behavior-a mini-review. Gerontology.

[84] Duca L., Blaise S., Romier B., Laffargue M., Gayral S., El Btaouri H., Maurice P. (2016). Matrix ageing and vascular impacts: Focus on elastin fragmentation. Cardiovasc. Res..

[85] Zaoui P., Cantin J.F., Alimardani–Bessette M., Monier F., Halimi S., Morel F., Cordonnier D. (2000). Role of metalloproteases and inhibitors in the occurrence and progression of diabetic renal lesions. Diabetes Metab..

